# Robust deep-learning based refrigerator food recognition

**DOI:** 10.3389/frai.2024.1442948

**Published:** 2024-12-04

**Authors:** Xiaoyan Dai

**Affiliations:** Advanced Technology Research Institute, Kyocera Corporation, Yokohama, Japan

**Keywords:** food recognition, deep learning, data augmentation, feature pyramid network, internet of Things, food management

## Abstract

Automatic food identification utilizing artificial intelligence (AI) technology in smart refrigerators presents an innovative solution. However, existing studies exhibit significant limitations. Achieving consistent high performance in recognition across varying camera distances and diverse real-world conditions remain a formidable challenge. Current approaches often struggle to accurately recognize items in scenarios involving occlusions, variable distortions, and complex backgrounds, thereby limiting their practical applicability in household environments. This study addresses these deficiencies by enhancing the Feature Pyramid Network (FPN) of YOLACT with an additional layer designed to capture nuanced information. Furthermore, we propose a two-stage data augmentation method that simulates diverse conditions including distortion and occlusion, to generate images that reflect various backgrounds and handheld scenarios. Comparative analyses with previous research and evaluations on our original dataset demonstrate that our approach significantly improves recognition rates for both typical and challenging real-world images. These enhancements contribute to more effective food waste management in households and indicate broader applications for automatic identification systems.

## 1 Introduction

Artificial intelligence (AI)-driven food recognition is an emerging technology aimed at identifying and classifying various types of food from images or videos. This technology leverages advanced AI and computer vision techniques, and can be used in various applications, such as dietary tracking, healthcare, and smart appliances.

Food waste is one of the most pressing challenges in contemporary society, carrying significant environmental, economic, and social implications. Alarmingly, approximately 30% of global food production is not consumed, resulting in the waste of valuable resources, increased greenhouse gas emissions, supply chain disruptions, and financial burdens for consumers. Many people unintentionally over-purchase food due to incomplete awareness of their refrigerator contents, often shopping without a well-organized list. To address this issue, smart refrigerators equipped with Internet of Things (IoT) technology offer a promising, cost-effective solution for more effective food inventory management.

**Figure 1:**
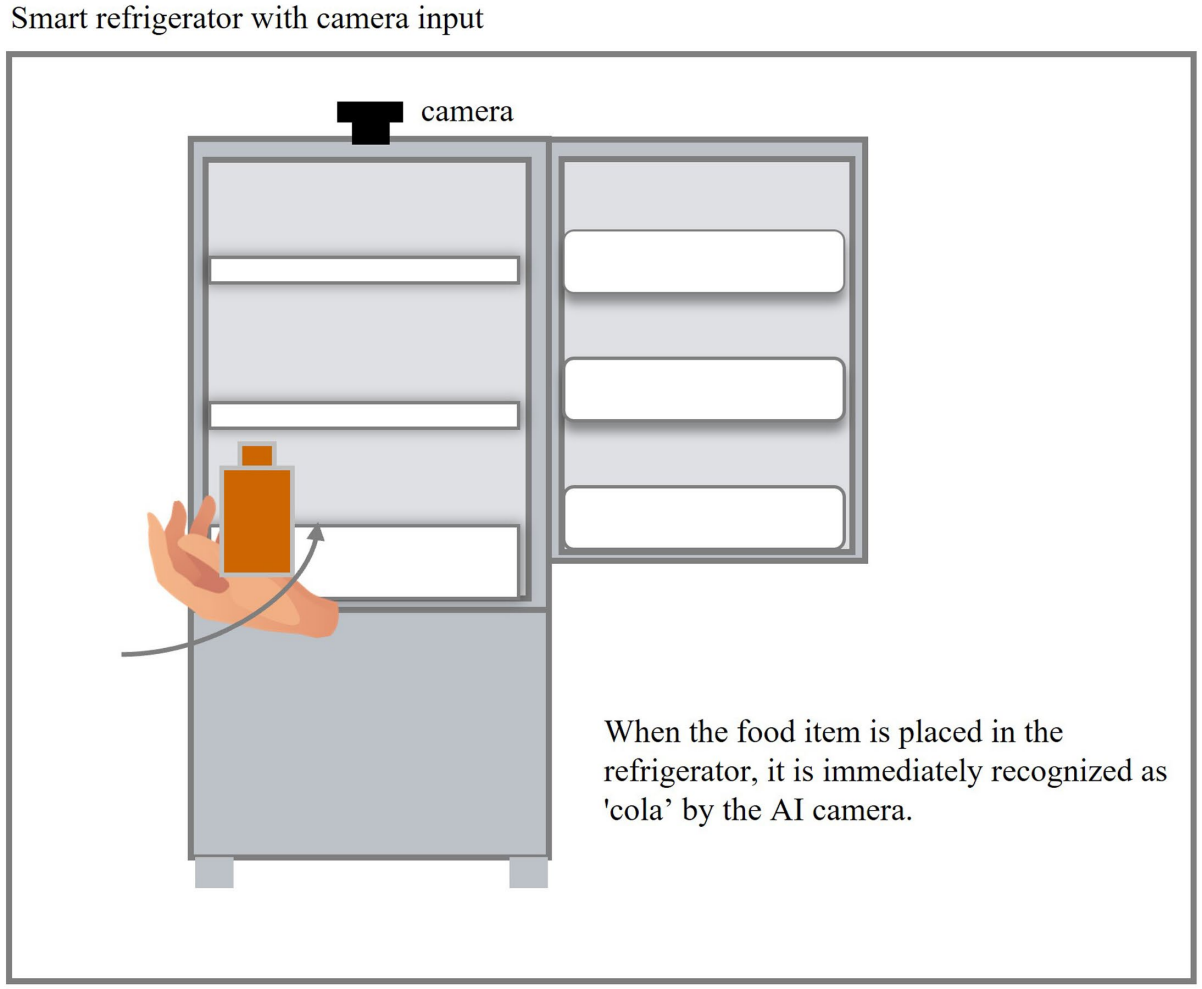
Images of a smart refrigerator with camera input.

This low-cost solution utilizes a single webcam to automatically recognize food items as they are placed into or taken out of the refrigerator. As illustrated in Figure 1, this solution has the potential to reduce unintentional over-purchasing, thereby minimizing food waste in household management.

A lot of research has been conducted on food recognition in smart refrigerators, which can be broadly categorized into scanning, sensing, and AI-driven approaches. Barcode scanning technology, as noted by Loh and Let (2004), Hong et al. (2007), Hossain and Abdelgawad (2018), and Dong et al. (2020), involves scanning the barcode tag of stored items to retrieve information. Radio Frequency Identification (RFID) scanning technology, highlighted by Shariff et al. (2019), Nejakar et al. (2020), and Jaipriya et al. (2021), employs identity tags attached to items. Sensing technologies, as demonstrated by Kale et al. (2015) and Goeddel et al. (2017), Nasir et al. (2018), Rezwan et al. (2018), Ahmed and Rajesh (2019), Kore et al. (2020), Huh et al. (2024), Gull and Bajwa (2021), Fakhrou et al. (2021), Banoth and Murthy (2024), employ weight, odor, or light sensors to provide real-time status updates on stored items. AI technologies, including machine learning, image recognition, and voice recognition, empower machines to emulate human decision-making processes. Various AI models have been employed in food recognition tasks, with Convolutional Neural Networks (CNNs) playing a crucial role in achieving accurate image-based classification. For example, Mezgec and Korouić (2017) demonstrated the effectiveness of CNNs in categorizing food images, and Lubura et al. (2022) used them to distinguish similar food types. Among CNNs, ResNet addresses challenges in deep networks, such as the vanishing gradient problem, and has been used successfully for food classification with complex datasets, as shown by Saha et al. (2020) and Bolya et al. (2021). Inception V3, another CNN architecture, efficiently captures spatial details, making it ideal for food recognition, as shown by Huang et al. (2019). YOLO, designed for real-time detection, is well-suited for dynamic food tracking in applications like restaurant ordering systems, as demonstrated by Xiao et al. (2023) and Rejin and Sambath (2024). MobileNet, optimized for mobile devices, enables food recognition by minimizing computational demands while retaining high accuracy, as shown by Sandler et al. (2018), Sahu et al. (2023), and Abiyev and Adepoju (2024).

Despite these advancements, challenges remain in food recognition technology. Current methods using barcode scanning cannot identify items without barcodes, such as fruits. RFID technology requires considerable manual effort for labeling, and sensing technologies often struggle with accurate differentiation of food items based solely on weight or odor. Although AI significantly enhances food recognition capabilities, many existing approaches are limited to recognizing items within a distance range of approximately 30 to 60 cm from the camera, which is considerably shorter than the typical usage distance in smart refrigerator applications. Furthermore, these methods often require frontal and close-up images, which do not account for the varied conditions encountered in real-world scenarios where items may be held at different angles and positions.

This research addresses these gaps and contributes to the advancement of AI-driven food recognition in practical applications. The key contributions include:

- The proposal of Feature Pyramid Network (FPN) designed to achieve high recognition performance across a broader range of camera distances, catering especially to small-sized products.
- The proposal of a novel data augmentation technique designed to enhance recognition performance in real-world images, even under conditions of distortion, occlusion, and variations in holding position, orientation, and background.

The remainder of this paper is organized as follows: Section 2 presents the proposed FPN and the data augmentation technique. Section 3 demonstrates the effectiveness of the proposed methods through extensive experimental evaluations. Finally, Section 4 concludes the paper and discusses potential future work.

## 2 Materials and methods

In this section, we will describe the proposed deep learning approach for automatic food recognition in smart refrigerators using camera input. We will first explain the proposed FPN and then discuss data augmentation.

### 2.1 AI model

We utilize YOLACT as the base of our object recognition model. YOLACT, an abbreviation for “You Only Look at Coefficients,” is a one-stage deep learning network designed to achieve real-time instance segmentation and object detection independently (Bolya et al., 2021). The YOLACT model’s training process uses a backbone network (e.g., ResNet) to extract image features, generates prototype masks, and combines them with learned coefficients to create instance-specific masks. It calculates the loss to update weights and minimize errors, optimizing through iterations and adjusting based on validation performance. We adopt this network and customize it to expand its capabilities. We have named our proposed model BroadFPN-YOLACT, as it combines YOLACT with our new FPN. The term ‘Broad’ highlights the network’s ability to capture features across a wide range of levels, emphasizing its comprehensive nature. This name will be used throughout the following sections.

#### 2.1.1 Architecture of BroadFPN-YOLACT

**Figure 2:**
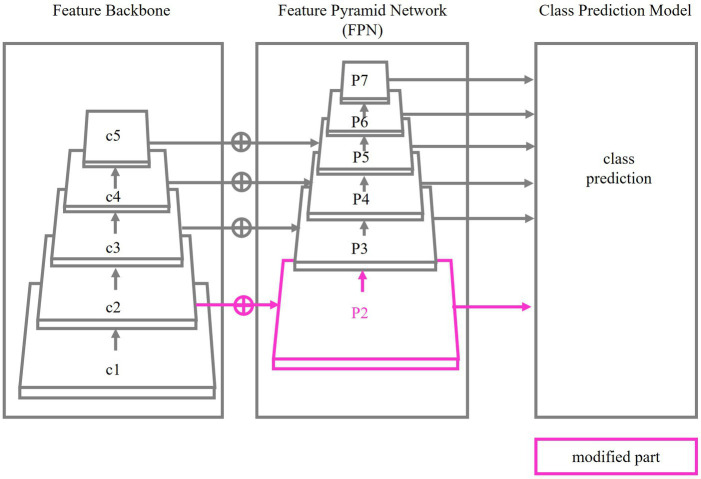
AI model structure.

The backbone network of BroadFPN-YOLACT is illustrated in Figure 2. It comprises three main components: Feature Backbone, FPN and Class Prediction Model. ResNet-101 (He et al., 2016) is employed as the default Feature Backbone, optimized for a base image resolution of 550 × 550 pixels. The Feature Backbone is responsible for extracting hierarchical features from input images, initiating with low-level features such as edges and textures and advancing to high-level semantic features such as object parts and categories. The FPN further refines these hierarchical features by capturing information at multiple scales, constructing a pyramid-like structure (from P3 to P7) where upper layers contain coarser features, while lower layers focus finer, detailed features. The Class Prediction Model is responsible for predicting the class label of the input image.

The resolution of each feature extraction layer of the FPN can be represented in the following Equation,


$$\mathrm{Resolution}\;\mathrm{at}\;\mathrm{layer}\;l=\frac{H}{2^{l}}$$

where H represents the input image size, and l denotes the layer index. The formula illustrates that layers closer to the network’s input preserve higher spatial resolution, allowing them to capture fine-grained details of objects, while deeper layers capture more abstract, high-level features, though at a lower resolution. In our object detection task, especially when dealing with small objects, it is critical that the network can capture fine details. Lower layers retain higher resolution features, which are particularly useful for this purpose. The formula mathematically justifies the spatial resolution differences between layers, showing a practical need to generate higher-resolution features from these lower layers to maintain both fine detail and broader context in object detection. To enhance the detection of small objects, we modified the FPN by adding a feature extraction layer, P2. This layer leverages high resolution features from earlier layers to improve detection accuracy for small objects. This modification, highlighted in pink in Figure 2, ensures that fine details crucial for detecting small objects are retained in the feature map, even if the input image is low resolution.

#### 2.1.2 Detail of BroadFPN-YOLACT

**Figure 3:**
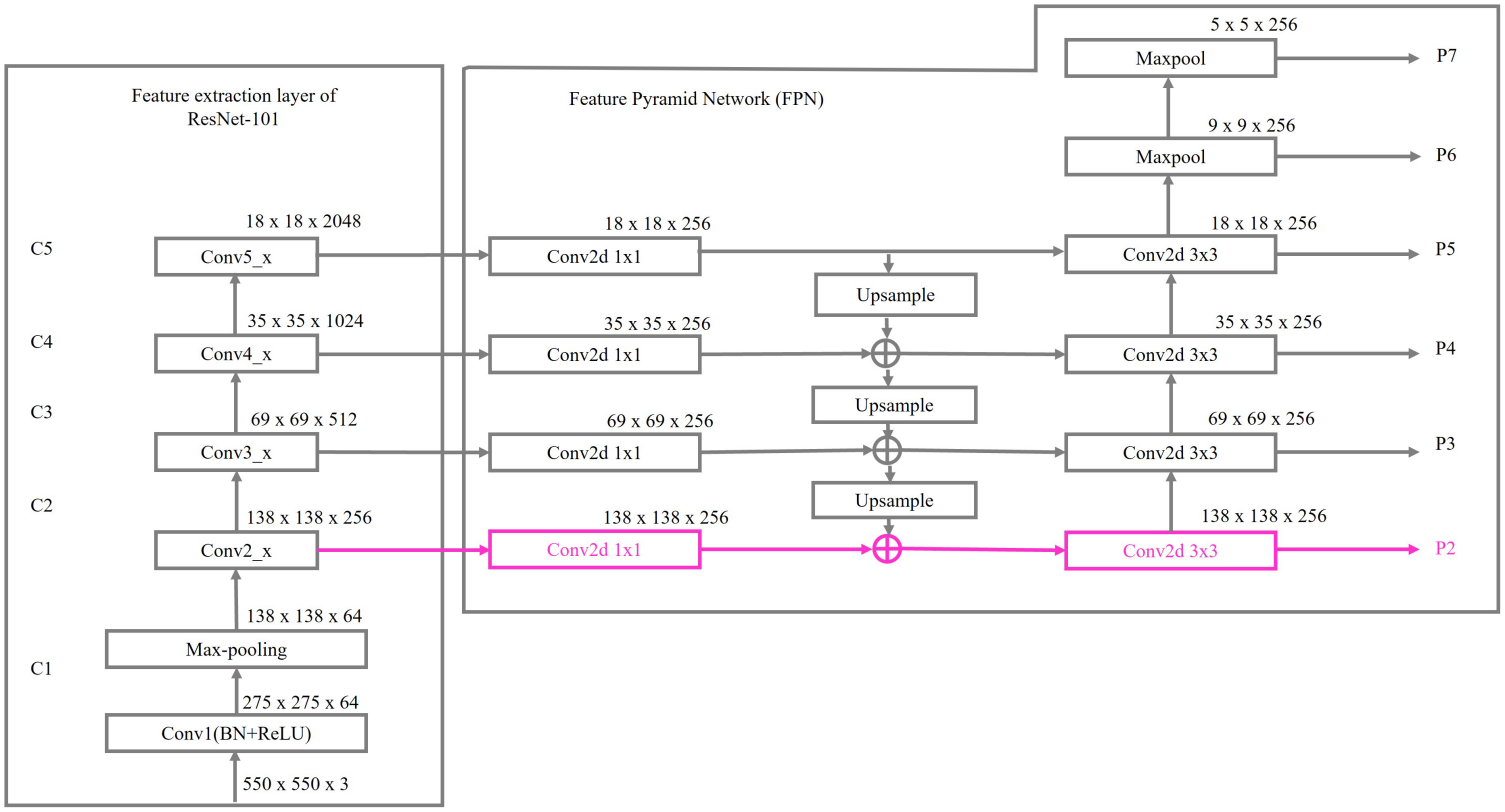
Details of BroadFPN-YOLACT.

Figure 3 illustrates the details of BroadFPN-YOLACT, highlighting an enhanced feature extraction layer P2, depicted in pink. The feature extraction layer of ResNet-101 consists of 101 layers organized into multiple stages. The initial convolutional layer, Conv1, employs a 7 × 7 filter with a stride of 2 and outputs 64 channels, primarily capturing fundamental edges and textures. Max-pooling subsequently reduces the spatial dimensions by half, down-sampling the feature maps. Thereafter, stages Conv2_x through Conv5_x produce feature maps at progressively different spatial resolutions, outputting 256, 512, 1,024, and 2048 channels respectively, with Conv5_x exhibiting the most abstract features and the smallest spatial resolution. These feature maps are then subjected to 1 × 1 convolutions, marking the starting points for the contraction of FPN.

Within the FPN, the top-down pathway progressively reconstructs higher-resolution feature maps by up-sampling from higher pyramid levels (e.g., C5) and merging them with lower-level feature maps (e.g., C4, C3, C2). Through lateral connections, the bottom-up pathway reinforces this top-down process, resulting in multi-scale feature maps (P3 to P7) that effectively incorporate high-level semantic and fine-grained spatial details across each scale.

To establish the additional layer P2, a 1 × 1 convolutional layer is added to the C2 feature map to reduce channel dimensions, and the top-down pathway is extended to reach the C2 layer. The P3 feature map is then up sampled and merged with the processed C2 feature map, thus creating P2. This enhanced multi-scale representation supports finer detail capture for smaller features, expands the range of effective scales and strengthens the network’s capacity for recognizing objects or features across varying sizes.

### 2.2 Data augmentation

**Figure 4:**
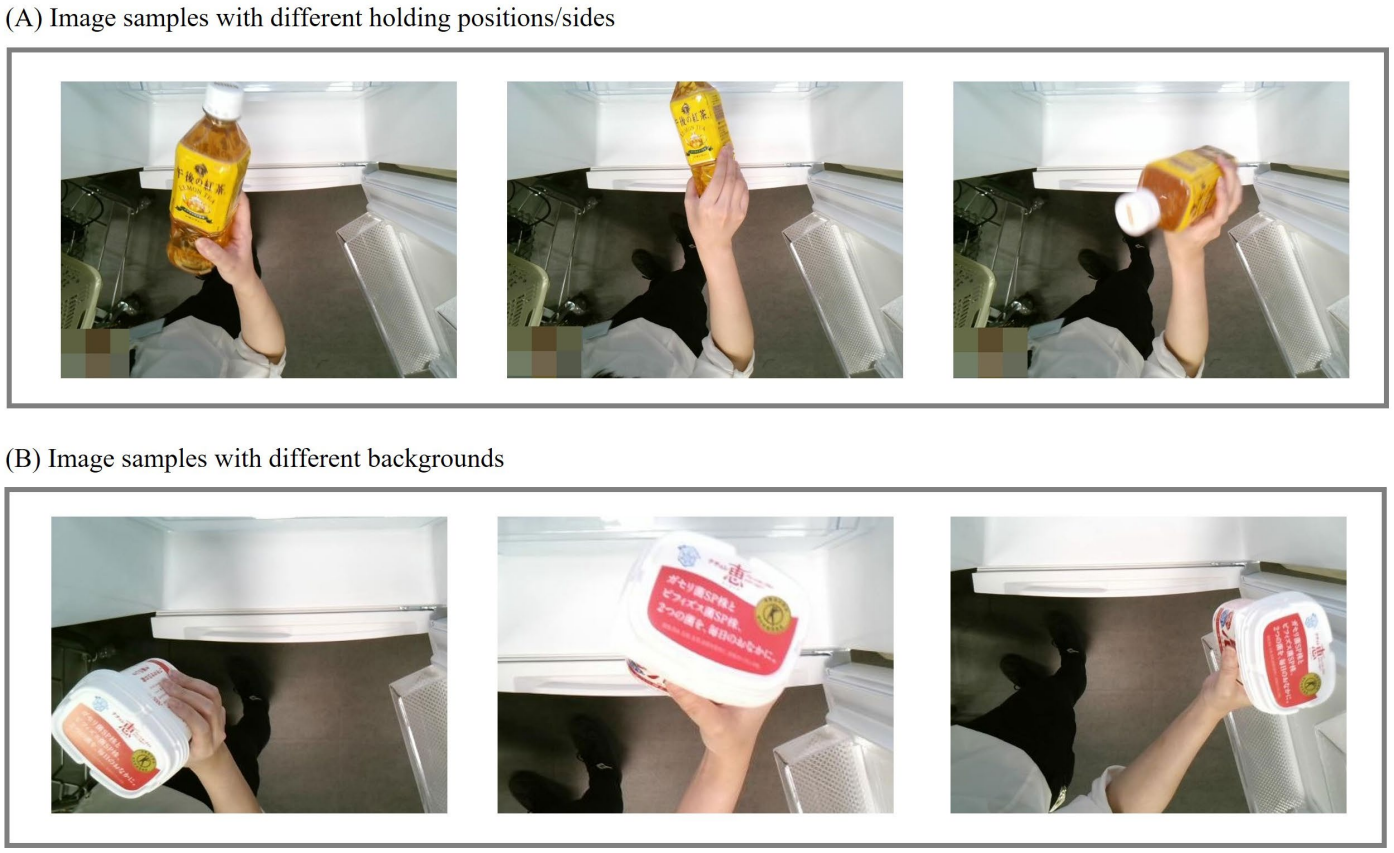
Image samples for food recognition in smart refrigerators. **(A)** Examples with different holding positions and sides; **(B)** Examples with different backgrounds.

Training deep learning models requires large datasets with ground truth annotations that closely resemble real-world scenarios. For food recognition in a smart refrigerator, various inputs need to be considered. As shown in Figure 4A, when placing or removing food from the refrigerator, users may handle it in different ways, potentially obscuring some parts of the food or altering its orientation relative to the camera. Additionally, as illustrated in Figure 4B, parts of the background, such as the refrigerator or the user’s body, may also be captured along with the food. These background elements can be variable.

In traditional data augmentation techniques, transformations such as scaling, rotation, color adjustments, and cropping are applied to the data to increase the dataset’s diversity. However, these augmentations often do not reflect the complexities of real-world environments accurately. This mismatch between artificially augmented data and actual, real-world data can limit the model’s performance, as the trained model may not generalize well to real-world scenarios.

Our proposed data augmentation approach is designed to bridge this gap by generating data that closely mimics real-world scenarios. We named our data augmentation Simu-Augmentation to suggest realistic, scenario-based transformations, combining the words ‘simulation’ and ‘augmentation’. This name will be used throughout the following sections.

**Figure 5:**
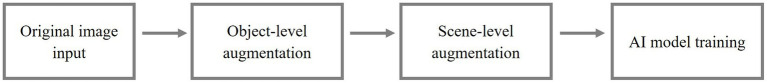
Processing flow for Simu-Augmentation.

With Simu-Augmentation, we aim to create a dataset that not only enhances model performance during training but also improves the model’s robustness when handling real-world input. The augmentation process, visualized in Figure 5, consists of two key stages. Each stage addresses different aspects of the image data to comprehensively enhance both object diversity and environmental variety, thus creating a dataset that more accurately represents realistic conditions.

1. Object-level augmentation: In the first stage, the focus is on creating variations within the objects themselves. This involves applying a range of transformations to simulate various object appearances with varying scales, distortions, blurs, lighting, orientations, and more. The stage generates a wide array of objects with diverse visual characteristics, ensuring that the dataset includes objects as they might appear in varied settings.
2. Scene-level augmentation: In the second stage, we incorporate these augmented objects into various simulated real-world scenes. Here, we position objects in different contexts and conditions to create a range of images that mimic handheld scenarios. This includes:

- Background variation: Placing objects against different backgrounds to reflect the diverse environmental contexts in which the model will encounter these objects.
- Handheld conditions: Simulating various handheld positions to account for natural variations in how users might capture or view objects in real-world situations.
- Compositional adjustments: Layering objects in realistic placements within the scene, ensuring consistency in lighting, relative object positioning, or else.

The final output of Simu-Augmentation is a set of images that not only contains a wide variety of object types and appearances but also contextual backgrounds that mirror realistic scenarios. This enriched dataset is then utilized for model training, helping the model generalize more effectively and achieve improved performance when applied to real-world data.

**Figure 6:**
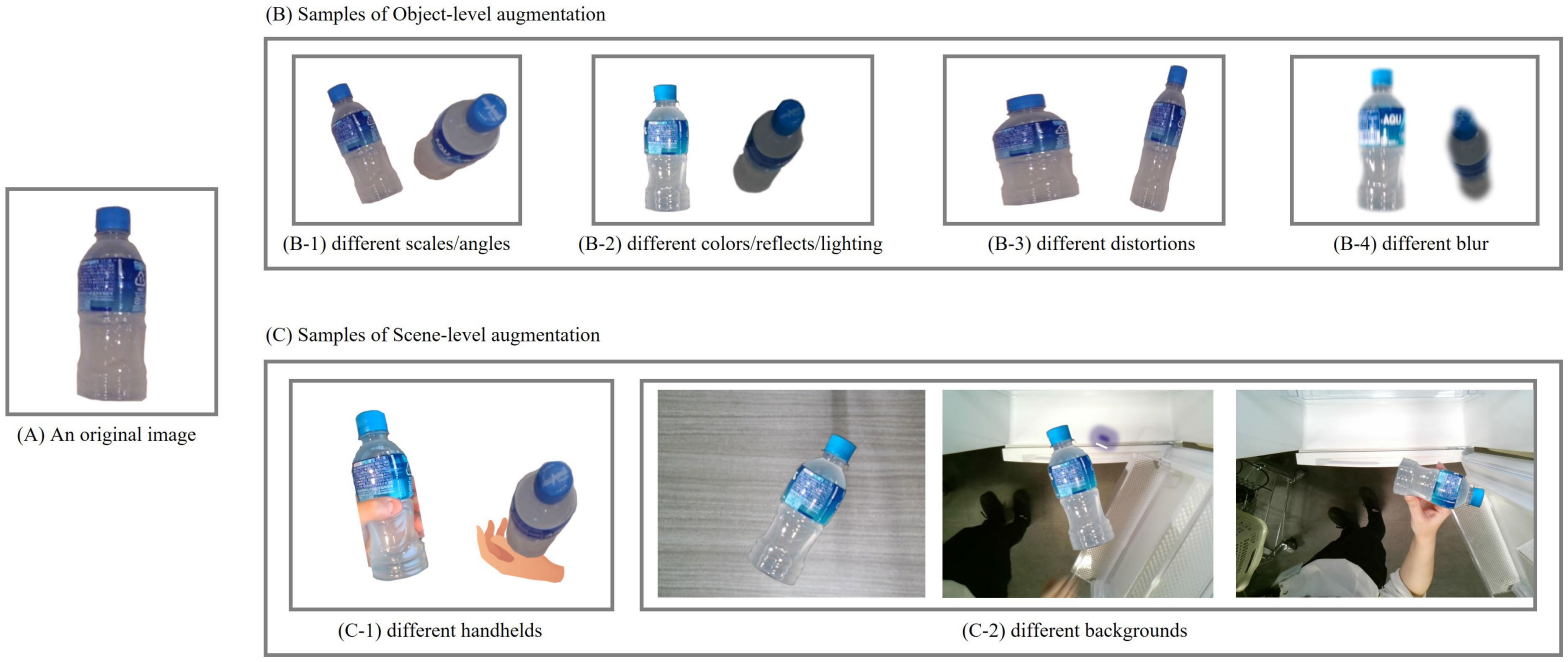
Sample of Simu-Augmentation. **(A)** An original image; **(B)** Examples of Object-level augmentation; **(C)** Examples of Scene-level augmentation.

Figure 6 illustrates examples of Simu-Augmentation. Panel (A) presents an original image. Panel (B) presents examples of Object-level augmentation, and Panel (C) demonstrates examples of Scene-level augmentation.

## 3 Results and discussion

In this section, we conduct a series of experiments to evaluate the effectiveness and robustness of the proposed approach for automatic food recognition in smart refrigerators. Our objective is to achieve high recognition accuracy for food items when they are placed in or removed from the refrigerator. As noted, objects may vary in size, the distances from the camera, and may exhibit distortions, reflections, or appear on various surfaces depending on how they are held. Since there is currently no existing dataset that encompasses these diverse conditions, we have developed a custom assessment dataset to reflect a wide range of input scenarios.

Three experiments were conducted to assess the performance of the proposed approaches, BroadFPN-YOLACT and Simu-Augmentation. Experiment 1 evaluates the recognition accuracy of BroadFPN-YOLACT in identifying objects that appear in low resolution due to small size or greater distance from the camera. We refer to this experiment as Recognition of objects in low resolution hereafter. Experiment 2 examines the effectiveness of Simu-Augmentation for enhancing recognition under diverse real-world conditions. We refer to this experiment as Recognition of objects under various conditions. Experiment 3 investigates the combined robustness of BroadFPN-YOLACT and Simu-Augmentation for recognizing a variety of food items under different conditions, including variations in object size, distance, lighting, handling orientation, and background complexity. We refer to this experiment as Recognition of various food items under different conditions. In the experiments, we employed a Full HD web camera (1920 × 1,080 pixels) with a 78° field of view to capture input images, and a GPU-equipped PC to perform recognition. Each time the refrigerator door is opened, the camera begins capturing video footage, sending images to the PC for AI-based food recognition. The identified information is then stored for future reference. We trained the model on an NVIDIA RTX 3090 GPU with 16GB of memory, using the PyTorch framework on the dataset over 50 epochs. The batch size was 8, and the initial learning rate was set to 0.0001, which was gradually reduced throughout the training. For object detection and recognition tasks, Precision, Recall, and F1-score are helpful metrics for assessing detection quality, while mean Average Precision (mAP) is widely used to evaluate the precision-recall trade-off, providing a comprehensive summary of performance. In our experiment, we use these metrics for evaluation, calculating mAP at a 50% Intersection over Union (IoU) threshold.

### 3.1 Recognition of objects in low resolution

As mentioned, the objective of this experiment is to evaluate the effectiveness of BroadFPN-YOLACT, for recognizing objects that appear in low resolution due to being far from the camera. Evaluation dataset 1 was constructed using over 30 types of small food items commonly found in stores, such as yogurt and beverage, with more than 100 images captured for each item under varied conditions. Given the complexity of the scenario, we employed data augmentation to prepare approximately 20,000 images per class to ensure diversity across different real-world scenarios. The images were split into a 90% training set and a 10% validation set. Each image was labeled with bounding boxes and class labels, and preprocessing steps included resizing to 550 × 550 pixels and normalization with a mean of 0.5 and a standard deviation of 0.5. As in prior AI approach, we utilized YOLACT with layers of FPN spanning P3 to P7, and YOLOv8, a one-stage object detection model that provides improvements in both accuracy and processing speed. The existing data augmentation techniques were applied in this experiment.

**Figure 7:**
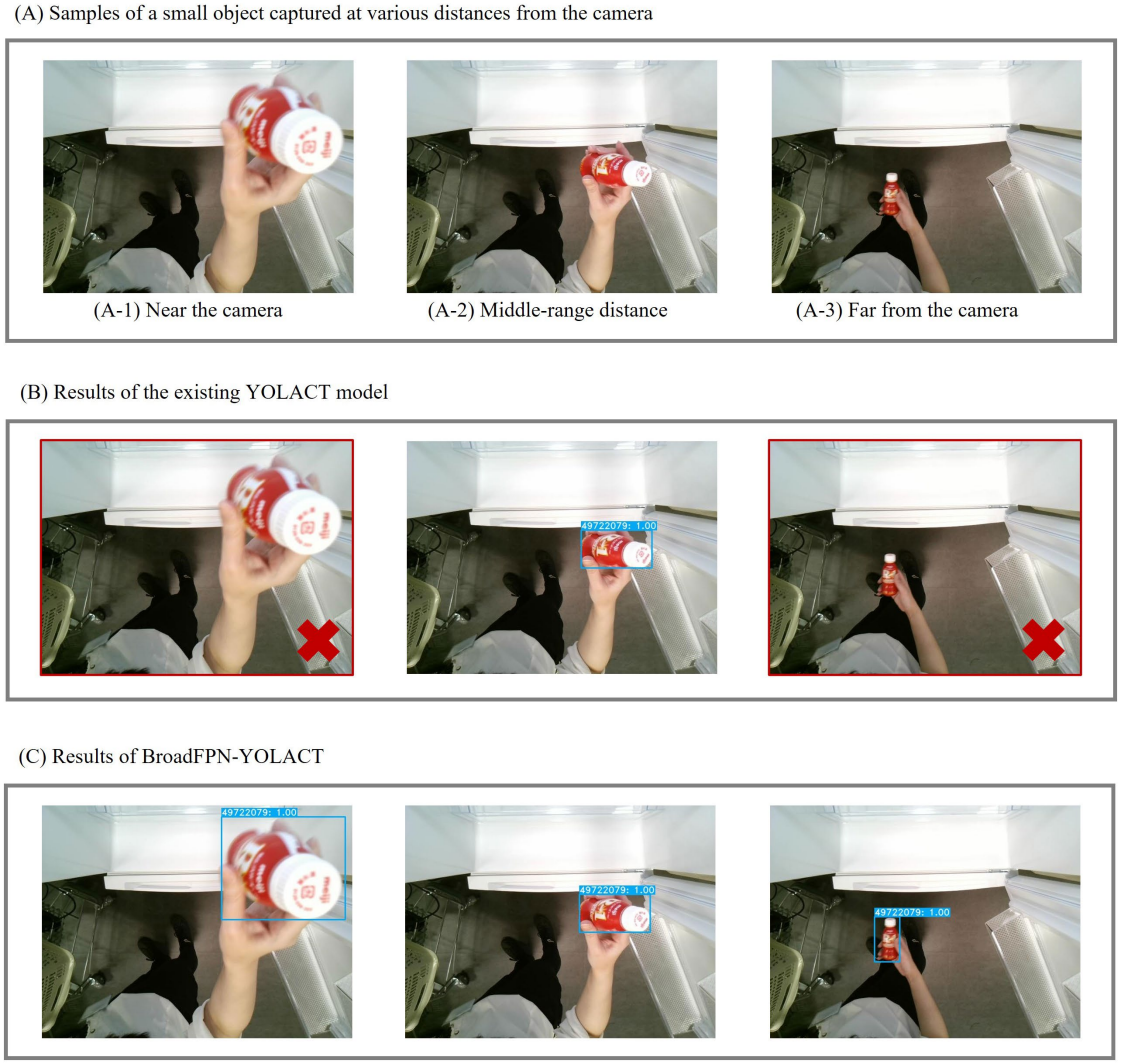
Sample results of Experiment 1. **(A)** Examples of a small object captured at various distances from the camera; **(B)** Results using the existing YOLACT model; **(C)** Results using the BroadFPN-YOLACT model.

Figure 7 presents sample images used in this experiment. In panel (A), three input images of a small beverage are shown at different distances from the camera: (A-1) within 20 cm, (A-2) at approximately 50 cm, and (A-3) at approximately 80 cm. Panel (B) illustrates the detection and recognition results of the existing YOLACT, while Panel (C) shows the results of the proposed BroadFPN-YOLACT. Blue rectangles indicate the detected regions, while white text on blue backgrounds represents recognition results, with confidence levels displayed as accompanying values. Images with different distortions challenge high detection accuracy, with images that were not recognized outlined in red rectangles. The results demonstrate that the existing approach struggled with recognition at closer and farther distances, while BroadFPN-YOLACT successfully recognized objects across a broader range of distances from the camera.

Table 1 presents the quantitative comparison results for these 30 types of small food items. To facilitate analysis, we categorized the distances from the camera into three ranges: less than 20 cm, from 20 to 60 cm, and from 60 to 100 cm. Distances exceeding 100 cm were not considered, given the typical height of refrigerators and humans. Experiments were conducted across various iterations to achieve an optimal balance between processing time and detection accuracy. The data highlighted in the blue rectangle of Table 1 corresponds to the results of Experiment 1, with outcomes from both YOLACT and the lightweight version YOLOv8n of YOLOv8. While YOLOv8n demonstrated satisfactory performance at standard distances (20–60 cm), BroadFPN-YOLACT yielded superior accuracy across all distance ranges. Specifically, in the 60–100 cm range, mAP improved markedly from 72.3 to 95.0% compared to YOLACT and from 90.0 to 95.0% compared to YOLOv8n. BroadFPN-YOLACT also achieved good results in Precision, Recall, and F1-score.

**Table 1:** Comparison of AI models and data augmentation approaches using dataset 1.

AI models			YOLACT	YOLOv8n	BroadFPN-YOLACT	YOLACT	YOLOv8n	BroadFPN-YOLACT
Data Augmentation			Existing Augmentation			Simu-Augmentation		
Distance to the camera	< 20 cm	Precision	0.91	0.93	0.94	0.92	0.94	0.96
		Recall	0.90	0.92	0.93	0.92	0.93	0.95
		F1-score	0.91	0.92	0.93	0.92	0.93	0.95
		mAP50	92.2%	93.8%	95.0%	93.5%	94.9%	96.6%
	20–60 cm	Precision	0.90	0.94	0.94	0.91	0.96	0.97
		Recall	0.89	0.93	0.94	0.91	0.95	0.95
		F1-score	0.89	0.93	0.94	0.91	0.95	0.96
		mAP50	91.7%	95.0%	95.1%	92.8%	96.3%	96.5%
	60–100 cm	Precision	0.78	0.88	0.94	0.81	0.91	0.97
		Recall	0.75	0.86	0.93	0.78	0.90	0.96
		F1-score	0.76	0.87	0.93	0.79	0.90	0.96
		mAP50	72.3%	90.0%	95.0%	76.1%	92.4%	96.9%

These results highlight the efficacy of BroadFPN-YOLACT for capturing finer details in small objects or those positioned farther from the camera. This experiment thus validated the model’s robustness in recognizing objects across varying distances.

### 3.2 Recognition of objects under various conditions

The objective of this experiment is to evaluate the effectiveness of the proposed data augmentation method, Simu-Augmentation, for recognizing images under real-world conditions. We utilized the same dataset developed for Experiment 1 and compared the performance of Simu-Augmentation against traditional data augmentation techniques using the same AI model. To isolate the impact of distance variations, we assessed performance separately across each distance range.

**Figure 8:**
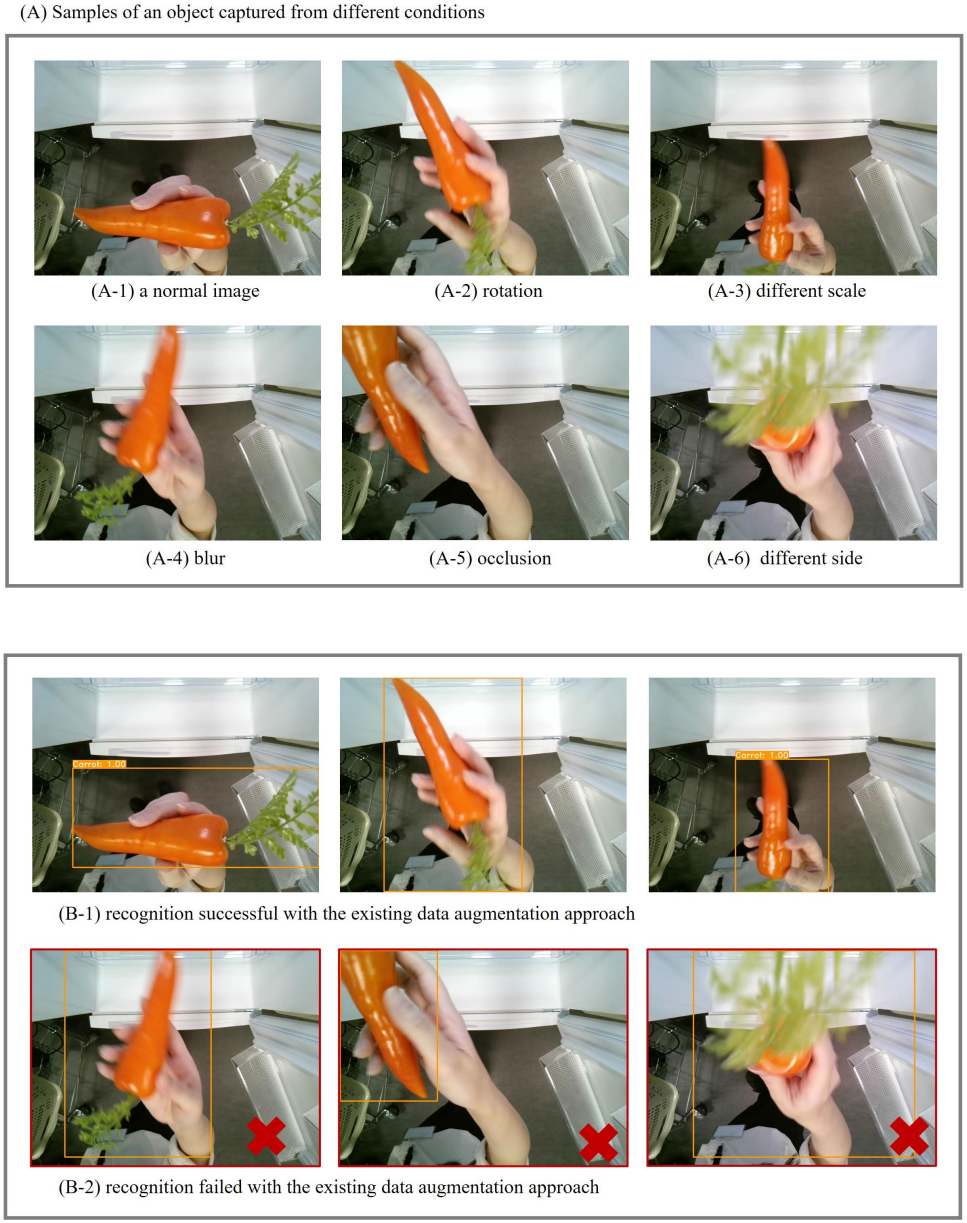
Sample results of Experiment 2. **(A)** Examples of an object captured under different conditions; **(B)** Results of Simu-Augmentation.

Figure 8 presents sample images used in this experiment. Panel (A) displays six images of the same object captured under various conditions: (A-1) represents a normal image, (A-2) shows a rotated view, (A-3) features a slightly altered scale, (A-4) is blurred, (A-5) shows partial occlusion due to a different holding position, and (A-6) displays different sides of the object, revealing an additional surface for processing. Panel (B) presents the detection and recognition results achieved using Simu-Augmentation. For comparison purposes, we refer to the results shown in Panel (B) to elucidate the performance of the conventional augmentation. Recognition failures associated with the conventional data augmentation are outlined in red. The results indicated that while the conventional method can successfully recognize images of the object from the same side, as shown in (B-1), it struggles with images exhibiting varying holding positions and surfaces as shown in (B-2). In contrast, Simu-Augmentation effectively recognizes images across these different conditions.

The data highlighted in the black rectangle of Table 1 corresponds to the results of Experiment 2. The results demonstrate that recognition performance across all AI models improved with Simu-Augmentation compared to existing approach across all distance ranges. For instance, in the 60 to 100 cm range, mAP of YOLACT increased from 72.3 to 76.1% with Simu-Augmentation, while BroadFPN-YOLACT showed an improvement from 95.0 to 96.9%. Simu-Augmentation also achieved good results in Precision, Recall, and F1-score.

These findings indicate that Simu-Augmentation is effective in generating training data that closely simulates real-world scenario within smart refrigerators. This experiment demonstrates the ability of Simu-Augmentation to reliably enhance recognition performance across various object appearances and backgrounds.

### 3.3 Recognition of various food items under different conditions

The objective of this experiment is to evaluate the robustness of integrating the proposed BroadFPN-YOLACT with the proposed Simu-Augmentation, for the recognition of various food items. In contrast to Dataset 1, which focused on small objects, we curated an evaluation image set, Dataset 2, which consists of over 80 types of food items, including side dish, soy milk, snacks, and vegetables. Some examples are mashed potatoes, Caesar salad, vanilla soy milk, dark chocolate, carrots, and more. These items come in various sizes and categories. The dataset contains more than 100 images for each item, collected under different conditions to capture a diverse range of sizes and types. For comparison purposes, we employed a combination of the existing YOLACT model with conventional data augmentation technique and YOLOv8n with the same augmentation method.

**Figure 9:**
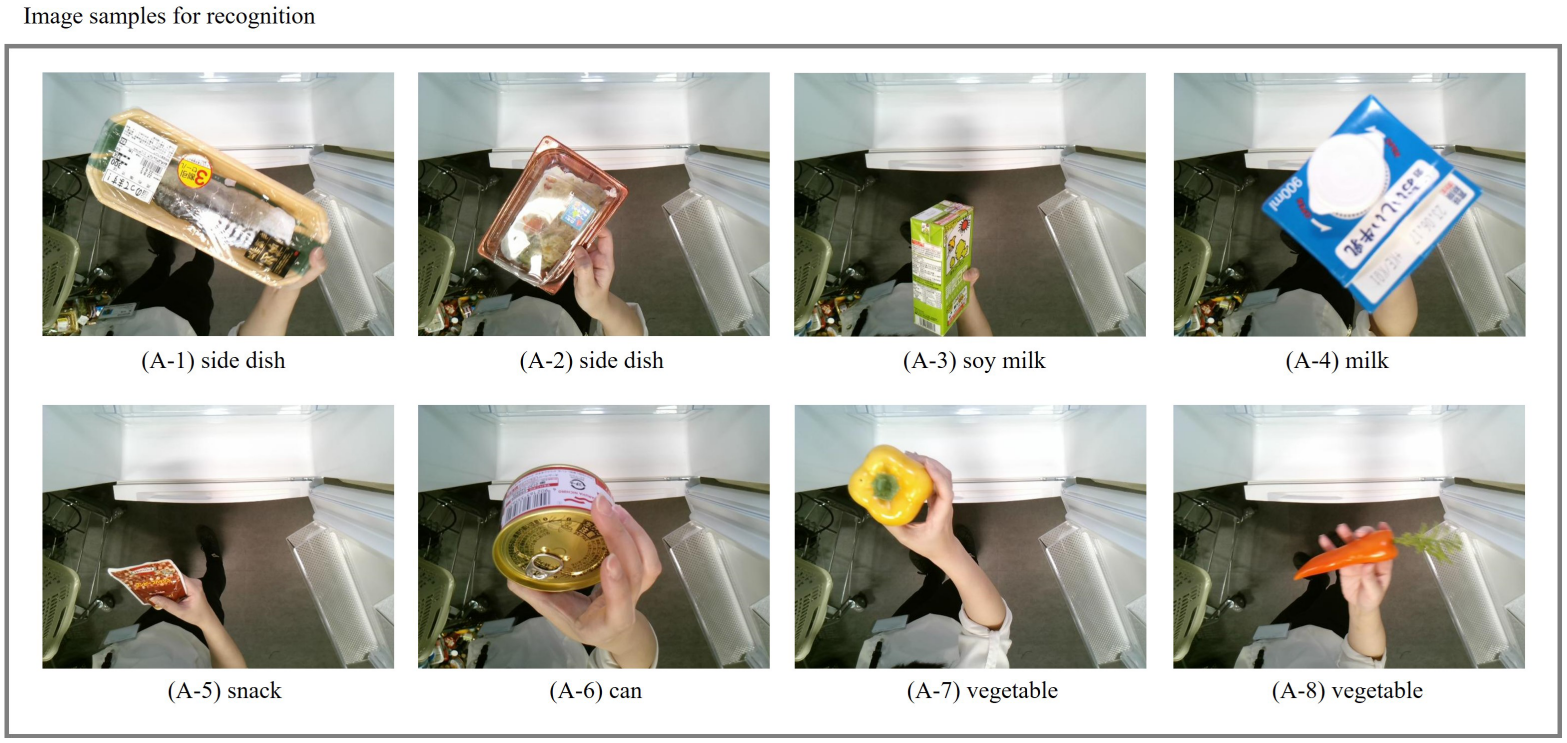
Samples images of Experiment 3.

Figure 9 presents sample images utilized in this experiment, featuring a variety of foods, including side dishes, drinks, and vegetables. These items can be positioned at varying distances from the camera, and their appearances were influenced by the way they are held.

**Figure 10:**
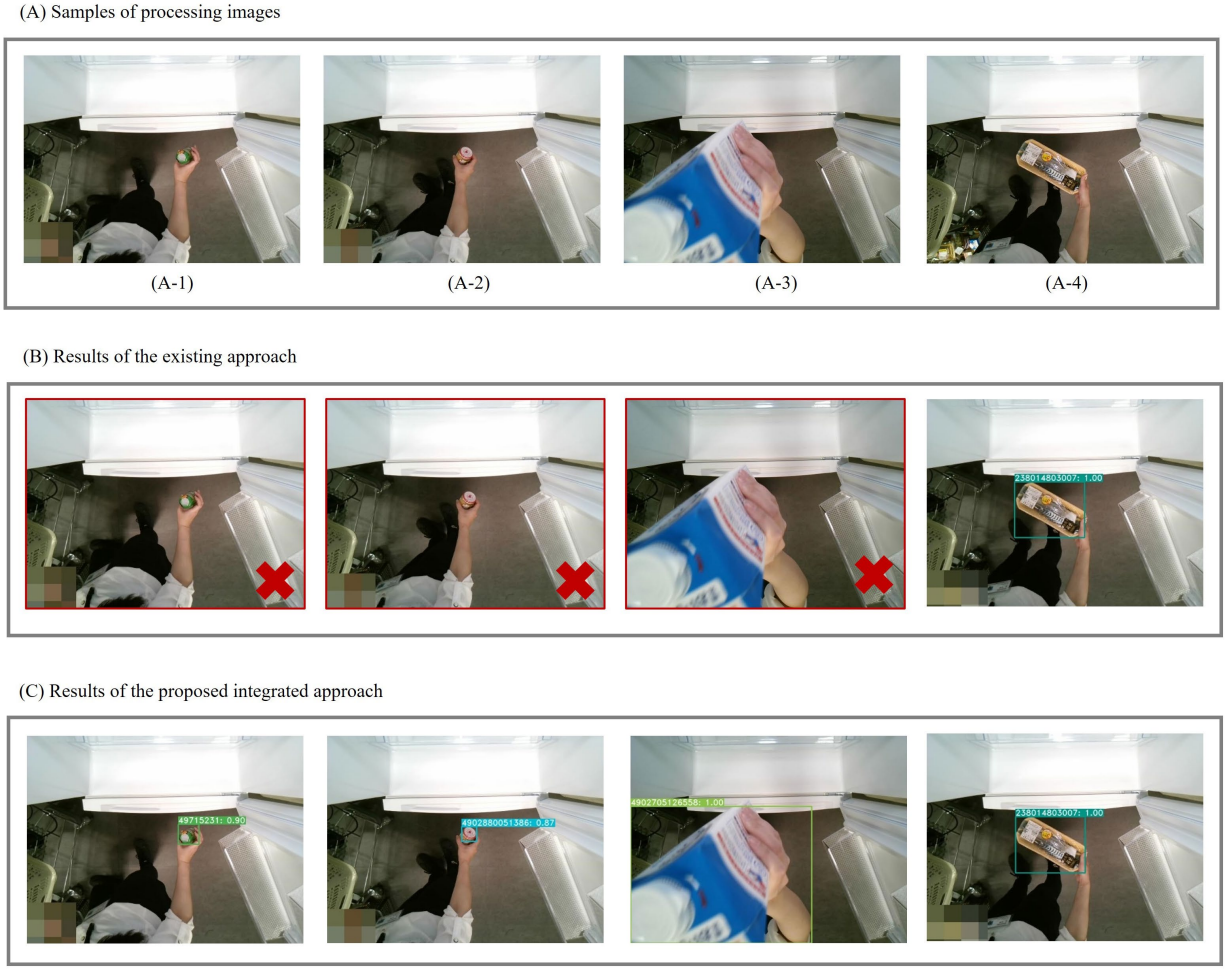
Sample results of Experiment 3. **(A)** Examples of processing images; **(B)** Results using the existing approach; **(C)** Results using the proposed integrated approach.

Figure 10 illustrates the comparative results of the two approaches. Panel (A) shows images of objects varying sizes and distances from the camera. Panels (B) and (C) display the results of conventional YOLACT approach and the proposed integrated approach, respectively. As depicted in Panel (B), the conventional approach exhibits challenges in recognizing small food items (A-1) and (A-2) and encounters difficulties with partially visible foods, as illustrated in (A-3). In contrast, the proposed approach yields satisfactory recognition results across all these cases.

We also quantitatively evaluate the improvement in recognition performance for these 80 food items across three distance ranges. As detailed in Table 2, the proposed approach consistently outperformed conventional methods across all distance ranges, achieving a maximum improvement of approximately 9.6% compared to the combination of YOLACT with the existing augmentation, and 2.4% compared to the combination of YOLOv8n with the existing augmentation in mAP. The integrated approach also achieved good results in Precision, Recall, and F1-score. Since the evaluation dataset includes both small and normal-sized objects, both the existing and proposed approaches demonstrate enhanced recognition performance compared to Experiment 1. However, our approach demonstrates superior recognition capabilities across a wider variety of objects.

**Table 2:** Comparison of AI models and data augmentation approaches using dataset 2.

AI model			YOLACT	YOLOv8n	BroadFPN-YOLACT
Data Augmentation			Existing Augmentation		Simu-Augmentation
Distance to the camera	<20 cm	Precision	0.94	0.95	0.97
		Recall	0.93	0.95	0.95
		F1-score	0.93	0.95	0.96
		mAP50	94.8%	96.2%	96.5%
	20–60 cm	Precision	0.93	0.97	0.98
		Recall	0.92	0.96	0.97
		F1-score	0.92	0.96	0.97
		mAP50	93.6%	97.0%	97.2%
	60–100 cm	Precision	0.85	0.93	0.97
		Recall	0.81	0.92	0.96
		F1-score	0.83	0.92	0.96
		mAP50	87.3%	94.5%	96.9%

## 4 Conclusion

Efforts to enable automatic food recognition in smart refrigerators have advanced, leveraging barcode scanning technology, RFID technology, sensing technologies, and AI-driven approaches. However, there are still significant challenges. Recognition systems using camera input often suffer from reduced accuracy at varying distances, especially for small or occlude objects. This is compounded by difficulties in handling real-world scenarios with diverse handholding positions, and cluttered backgrounds.

To address these issues, we propose a deep learning solution for robust, automatic food recognition using a standard webcam placed inside or outside the refrigerator. Unlike traditional sensor-based systems, our camera-based approach captures and analyzes images of items as they are placed into or removed from the refrigerator. By using YOLACT as the object detection network and enhancing the FPN and data augmentation, our system can detect objects at different distances and sizes with improved accuracy.

The key Contributions are

1. Enhanced feature extraction: We modify the FPN to better capture fine-grained details for small or distant items.
2. Novel data augmentation: Our data augmentation method creates diverse images simulating real-world distortions, variations in handholding, and backgrounds, improving robustness.
3. Comprehensive Dataset: Our custom dataset includes a range of objects, sizes, and conditions, allowing for more thorough testing and training.

Our experiments show that this method surpasses existing technologies, accurately recognizing various food items, from small to large, and adapting to various distances and positioning scenarios. This suggests potential applications in home settings where such factors frequently vary.

While promising, our current system focuses primarily on store-bought products with barcodes. Moreover, it lacks direct functionality for interpreting expiry dates, which could be critical for further usability in household food management.

Looking ahead, we aim to expand recognition capabilities to develop algorithms to recognize homemade foods and items without barcodes. We also aim to realize automatic expiry detection by incorporating a mechanism to identify expiry dates, further enhancing food management and waste reduction.

We believe this approach holds substantial promise for improving home food management, reducing food waste, and conserving energy. With further development, it could become a versatile technology applicable across numerous domains, from home kitchens to commercial inventory systems.

## Data Availability

The raw data supporting the conclusions of this article will be made available by the authors, without undue reservation.
